# Supplementary material for: Modeling glioblastoma heterogeneity as a dynamic network of cell states
Source: Mol Syst Biol. 2021 Sep 16;17(9):e10105. doi: 10.15252/msb.202010105 (PMC8444284; doi:10.15252/msb.202010105)
Supplement: Supplementary file 6 — Source Data for Figure 5 [file MSB-17-e10105-s004.zip › Figure5A_sourcedata/GSEA_3071/hallmarks_stateVI.GseaPreranked.1623742557031/HALLMARK_ESTROGEN_RESPONSE_LATE.html]

Details for gene set HALLMARK\_ESTROGEN\_RESPONSE\_LATE[GSEA]

|  || Dataset | state63071 |
| Phenotype | NoPhenotypeAvailable |
| Upregulated in class | na\_neg |
| GeneSet | HALLMARK\_ESTROGEN\_RESPONSE\_LATE |
| Enrichment Score (ES) | -0.35255903 |
| Normalized Enrichment Score (NES) | -1.6032463 |
| Nominal p-value | 0.043165468 |
| FDR q-value | 0.15498275 |
| FWER p-Value | 0.734 |
Table: GSEA Results Summary

  

Fig 1: Enrichment plot: HALLMARK\_ESTROGEN\_RESPONSE\_LATE      
 Profile of the Running ES Score & Positions of GeneSet Members on the Rank Ordered List

  

| PROBE | GENE SYMBOL | GENE\_TITLE | RANK IN GENE LIST | RANK METRIC SCORE | RUNNING ES | CORE ENRICHMENT || 1 | GAL |  |  | 2 | 1.442 | 0.0371 | No |
| 2 | CAV1 |  |  | 13 | 1.087 | 0.0626 | No |
| 3 | CDC20 |  |  | 15 | 1.073 | 0.0903 | No |
| 4 | PERP |  |  | 27 | 0.969 | 0.1125 | No |
| 5 | PRSS23 |  |  | 108 | 0.732 | 0.1093 | No |
| 6 | FLNB |  |  | 120 | 0.709 | 0.1247 | No |
| 7 | MYOF |  |  | 157 | 0.655 | 0.1317 | No |
| 8 | FKBP4 |  |  | 279 | 0.550 | 0.1122 | No |
| 9 | GALE |  |  | 355 | 0.510 | 0.1046 | No |
| 10 | XBP1 |  |  | 389 | 0.494 | 0.1082 | No |
| 11 | IGFBP4 |  |  | 571 | 0.433 | 0.0689 | No |
| 12 | PAPSS2 |  |  | 600 | 0.425 | 0.0722 | No |
| 13 | TFPI2 |  |  | 727 | 0.391 | 0.0472 | No |
| 14 | FOXC1 |  |  | 927 | 0.357 | 0.0009 | No |
| 15 | KIF20A |  |  | 961 | 0.351 | 0.0008 | No |
| 16 | PPIF |  |  | 983 | 0.348 | 0.0040 | No |
| 17 | SLC7A5 |  |  | 989 | 0.348 | 0.0117 | No |
| 18 | ATP2B4 |  |  | 1092 | 0.331 | -0.0082 | No |
| 19 | TSPAN13 |  |  | 1195 | 0.317 | -0.0284 | No |
| 20 | SORD |  |  | 1239 | 0.311 | -0.0323 | No |
| 21 | MEST |  |  | 1294 | 0.304 | -0.0395 | No |
| 22 | DYNLT3 |  |  | 1472 | 0.283 | -0.0816 | No |
| 23 | UGDH |  |  | 1548 | 0.274 | -0.0954 | No |
| 24 | FDFT1 |  |  | 1552 | 0.274 | -0.0891 | No |
| 25 | MOCS2 |  |  | 1617 | 0.267 | -0.1000 | No |
| 26 | ISG20 |  |  | 1756 | 0.255 | -0.1319 | No |
| 27 | DNAJC1 |  |  | 1848 | -0.256 | -0.1507 | No |
| 28 | MAPT |  |  | 1922 | -0.267 | -0.1641 | No |
| 29 | IMPA2 |  |  | 2078 | -0.291 | -0.1998 | No |
| 30 | NCOR2 |  |  | 2134 | -0.303 | -0.2073 | No |
| 31 | CPE |  |  | 2173 | -0.309 | -0.2098 | No |
| 32 | TIAM1 |  |  | 2303 | -0.334 | -0.2372 | No |
| 33 | STIL |  |  | 2376 | -0.349 | -0.2482 | No |
| 34 | TFF3 |  |  | 2670 | -0.416 | -0.3193 | No |
| 35 | RPS6KA2 |  |  | 2709 | -0.428 | -0.3187 | No |
| 36 | RAB31 |  |  | 2806 | -0.459 | -0.3336 | No |
| 37 | OLFM1 |  |  | 2875 | -0.485 | -0.3399 | Yes |
| 38 | SLC26A2 |  |  | 2883 | -0.489 | -0.3291 | Yes |
| 39 | IDH2 |  |  | 2966 | -0.524 | -0.3384 | Yes |
| 40 | CDC6 |  |  | 2990 | -0.534 | -0.3309 | Yes |
| 41 | PDLIM3 |  |  | 3011 | -0.546 | -0.3222 | Yes |
| 42 | ID2 |  |  | 3026 | -0.554 | -0.3117 | Yes |
| 43 | COX6C |  |  | 3054 | -0.565 | -0.3045 | Yes |
| 44 | FABP5 |  |  | 3079 | -0.577 | -0.2961 | Yes |
| 45 | GINS2 |  |  | 3119 | -0.598 | -0.2914 | Yes |
| 46 | CELSR2 |  |  | 3121 | -0.598 | -0.2761 | Yes |
| 47 | HOMER2 |  |  | 3122 | -0.598 | -0.2605 | Yes |
| 48 | BTG3 |  |  | 3184 | -0.647 | -0.2607 | Yes |
| 49 | RBBP8 |  |  | 3189 | -0.652 | -0.2448 | Yes |
| 50 | ALDH3A2 |  |  | 3213 | -0.683 | -0.2334 | Yes |
| 51 | FARP1 |  |  | 3275 | -0.734 | -0.2313 | Yes |
| 52 | CD9 |  |  | 3445 | -0.995 | -0.2526 | Yes |
| 53 | SLC29A1 |  |  | 3449 | -1.005 | -0.2272 | Yes |
| 54 | EMP2 |  |  | 3456 | -1.020 | -0.2022 | Yes |
| 55 | TOP2A |  |  | 3457 | -1.020 | -0.1756 | Yes |
| 56 | CHPT1 |  |  | 3470 | -1.048 | -0.1516 | Yes |
| 57 | FOS |  |  | 3476 | -1.056 | -0.1255 | Yes |
| 58 | NBL1 |  |  | 3535 | -1.240 | -0.1094 | Yes |
| 59 | CKB |  |  | 3579 | -1.575 | -0.0803 | Yes |
| 60 | ASCL1 |  |  | 3604 | -1.822 | -0.0395 | Yes |
| 61 | CXCL14 |  |  | 3605 | -1.867 | 0.0092 | Yes |
Table: GSEA details [plain text format]

  

Fig 2: HALLMARK\_ESTROGEN\_RESPONSE\_LATE: Random ES distribution      
 Gene set null distribution of ES for **HALLMARK\_ESTROGEN\_RESPONSE\_LATE**
